# Targeting TOP2A in Ovarian Cancer: Biological and Clinical Implications

**DOI:** 10.3390/curroncol31120594

**Published:** 2024-12-20

**Authors:** Fulvio Borella, Stefano Fucina, Ylenia Seminara, Pietro Denti, Domenico Ferraioli, Luca Bertero, Niccolò Gallio, Jessica Cusato, Giorgio Valabrega, Alberto Revelli, Luca Marozio, Stefano Cosma

**Affiliations:** 1 Gynecology and Obstetrics 1U, Department of Surgical Sciences, University of Turin, 10126 Turin, Italy; stefano.fucina@unito.it (S.F.); ylenia.seminara@unito.it (Y.S.); pietro.denti@unito.it (P.D.); luca.marozio@unito.it (L.M.); stefano.cosma@unito.it (S.C.); 2 Department of Gynecology, Léon Bérard, Comprehensive Cancer Centre, 69008 Lyon, France; domenico.ferraioli@lyon.unicancer.fr; 3 Pathology Unit, Department of Medical Sciences, University of Turin, 10126 Turin, Italy; luca.bertero@unito.it; 4 Gynecology and Obstetrics 2U, Department of Surgical Sciences, University of Turin, 10126 Turin, Italy; niccolo.gallio@edu.unito.it (N.G.); alberto.revelli@unito.it (A.R.); 5 Laboratory of Clinical Pharmacology and Pharmacogenetics, Department of Medical Sciences, University of Turin, 10149 Turin, Italy; jessica.cusato@unito.it; 6 Department of Oncology, University of Turin, Medical Oncology, Ordine Mauriziano Hospital, 10128 Turin, Italy; giorgio.valabrega@unito.it

**Keywords:** TOP2A, topoisomerase, ovarian cancer, anthracyclines, doxorubicin, etoposide, immune checkpoint inhibitors, PARPi

## Abstract

The enzyme topoisomerase II alpha (TOP2A) plays a critical role in DNA replication and cell proliferation, making it a promising target for cancer therapy. In epithelial ovarian cancer (EOC), TOP2A overexpression is associated with poor prognosis and resistance to conventional treatments. This review explores the biological functions of TOP2A in EOC and discusses its potential as a therapeutic target. We highlight studies on the mechanisms through which TOP2A contributes to tumor progression and recurrence. Additionally, we evaluate the clinical implications of targeting TOP2A, including the use of TOP2A inhibitors and their combination with novel drugs. We provide a comprehensive overview of the current understanding and future directions for targeting TOP2A in the management of EOC.

## 1. Introduction

Ovarian cancer (OC) remains one of the leading causes of death among gynecologic cancers. In 2022, about 324,398 women worldwide received a new diagnosis of OC and 206,839 women will have died due to this disease [1].

Despite progress in the knowledge of the molecular biology of EOC and new treatment options, the five-year survival rate has remained relatively unchanged [2].

Between 60% and 70% of women are diagnosed with International Federation of Gynecology and Obstetrics (FIGO) stages III-IV. Although many OC patients initially respond to treatment, most of them experience recurrence or tumor progression. The standard treatment approach includes surgical removal and cytoreduction. The presence of visible residual tumor after debulking surgery remains one of the most significant prognostic factors. Post-operative treatment typically involves a combination of carboplatin and paclitaxel administered every three weeks for six cycles. Adding the antiangiogenic vascular endothelial growth factor inhibitor (VEGF) bevacizumab to platinum-based doublets and continuing maintenance therapy in advanced stages have shown a modest improvement in progression-free survival (PFS) (10.5 months with standard therapy vs. 15.9 months with bevacizumab) [2,3,4].

Multiple studies have assessed the effectiveness of poly ADP ribose polymerase enzyme inhibitors (PARPis), leading to their recent approval for treating epithelial ovarian cancer (EOC). Olaparib is approved for BRCA-mutated patients as a maintenance therapy following first-line chemotherapy and for platinum-sensitive relapse. Instead, Niraparib and Rucaparib are approved for maintenance therapy or platinum-sensitive relapse regardless of BRCA status.

Currently, PARPis have been shown to improve PFS. However, to date, no significant impact on overall survival (OS) has been observed with these drugs [5,6,7,8].

The choice of further therapies for recurrent disease will depend on the platinum-free interval (the time between the last platinum-based treatment and the detection of disease progression), the BRCA mutation status, and the patients’ characteristics.

For patients with partially platinum-sensitive disease, who progress between 6 and 12 months after first-line platinum therapy, standard treatments include second-line platinum-based doublet chemotherapy or a combination of pegylated liposomal doxorubicin (PLD) and trabectedin [2]. For those with platinum-resistant epithelial ovarian cancer (EOC), progressing within six months of their last platinum therapy, treatment options are limited and often debated.

Subsequent treatments with non-platinum single-agent chemotherapies typically show limited effectiveness [2]. Recently, promising results have been observed in a subgroup of patients with folate receptor alpha-positive, platinum-resistant EOC, who had previously received one to three systemic treatment regimens and were treated with mirvetuximab soravtansine-gynx (a folate receptor alpha-directed antibody–drug conjugate) combined with a maytansinoid microtubule inhibitor [9]. The most common cytotoxic drugs used in the platinum-resistant setting are PLD, paclitaxel, topotecan, and gemcitabine. PLD is often preferred due to its favorable toxicity profile [10,11,12,13]. PLD targets the TOP2A gene, located at the q21 locus on chromosome 17, near the HER2 gene, which encodes the nuclear enzyme type 2 topoisomerase alpha (TOP2A), essential for maintaining DNA stability. This review aims to summarize the clinical and biological implications of targeting TOP2A, with a focus on treating EOC.

## 2. Topoisomerase Function

The DNA replication and RNA transcription processes create physiologically positive (corresponding to over-twisting of the helix) and negative (corresponding to under-twisting of the helix) DNA supercoiling [14].

The enzymes that rule the state of the genetic material are called topoisomerases. Indeed, these enzymes remove the DNA supercoils to prevent excess supercoiling and the breakdown of the replication machinery.

Catenated DNA rings can be formed if two replication forks are composed of interwound daughter molecules. Thus, precatenate DNA is converted to catenates. Removing the rings is called decatenation; during this process, one of the DNA rings is cleaved and the other is passed through the double-strand break [15,16,17].

The DNA topoisomerases are divided into two different classes [18].

Type I topoisomerases: these enzymes define the DNA topology by utilizing an active site with a tyrosine residue to cut one strand of the DNA. This allows a single DNA strand to pass through the break in the opposite strand, forming a phosphodiester bond with the protein. Unlike type II topoisomerases, type I does not rely on ATP hydrolysis to function; instead, it derives its energy from the intrinsic strain energy of the supercoiled DNA.

Type II topoisomerases: in contrast, type II topoisomerases induce a double-strand break using a similar active site with tyrosine residues. This allows another double-stranded DNA segment to pass through the break. Unlike type I, type II topoisomerases are ATP-dependent and have a separate ATP-binding domain apart from the DNA-binding domain.

Topoisomerase II functions by forming complexes with DNA, which are essential for proper chromosome separation. If there are too few complexes, cells fail in mitosis and die. Conversely, an excess of these complexes can lead to permanent double-stranded breaks, triggering repair pathways and causing DNA abnormalities like chromosome translocations. If these breaks are too numerous, they result in cell death.

Based on the mechanism of action, the polarity of the DNA–protein bond, and the number of overhanging DNA bases, topoisomerases are classified into IA, IB, and IIA [19]. In particular, TOP2A derives from a gene located on 17q21.2. This gene is up-regulated in proliferating cells and represents a worthy pharmacologic target for some anti-cancer drugs, especially for managing recurrent EOC. These anti-cancer drugs’ mechanism of action consists of stabilizing a transient covalent enzyme–DNA complex, which produces DNA strand cleavage and apoptosis [20,21,22].

## 3. The Role of TOP2A in Carcinogenesis

It is now well established that chromosome instability can lead to tumorigenesis, suggesting that abnormal mitosis plays a role in promoting tumor evolution. The process of mitosis is regulated and coordinated by multiple pathways to ensure accurate DNA replication. During mitosis, chromatids condense, and sister chromatids are pulled to opposite sides of the cell, ensuring equal distribution of duplicated DNA among daughter cells.

TOP2A is crucial in the decatenation checkpoint; defects in this checkpoint can lead to chromosomal missegregation. Additionally, deficiencies in the decatenation checkpoint may result in further chromosome imbalances in tumor cells, increasing cancer aggressiveness [23,24].

The expression, genetic alteration, and enzyme activity of TOP2A have been studied extensively, and its prognostic value has been highlighted by numerous studies across various malignancies. For instance, in studies of patients with hepatocellular carcinoma, Wong et al. [25] found that TOP2A expression correlated with advanced histological grading (*p* ≤ 0.001), microvascular invasion (*p* = 0.004), and early onset of malignancy (*p* = 0.007). Furthermore, elevated TOP2A expression was closely associated with poor OS (*p* = 0.003) and disease-free survival (DFS) (*p* < 0.0001) [17].

Elevated TOP2A expression has been observed in several solid tumors, and its overexpression is significantly correlated with an aggressive tumor phenotype, advanced disease stage, tumor recurrence, and poor survival [24,26].

In breast cancer, TOP2A overexpression is associated with higher tumor grade and Ki67 index [27]. Other authors reported similar results: increased TOP2A expression is particularly evident in more aggressive subtypes of early breast cancer [28].

The expression of TOP2A can also be influenced by p53. In p53-deficient cells, TOP2A-DNA complexes accumulate, leading to DNA damage during replication. This suggests that p53 prevents DNA topological stress during the S phase, ensuring normal replication fork progression. Inhibition of transcription in p53-deficient cells mitigates DNA damage and decreases TOP2A-DNA complexes, thus restoring cell viability [29].

## 4. TOP2A in Ovarian Cancer

High-grade serous EOC is marked by numerous molecular abnormalities and mutations, with TP53 being mutated in nearly all instances [30,31,32,33]. The carcinogenesis of high-grade serous EOC also involves somatic or germline mutations in homologous recombination genes like BRCA1 and BRCA2 [30,32]. Furthermore, high-grade serous EOC exhibits extensive copy number alterations [31,33] and it is influenced by pathways such as FXM1, Rb1, PI3K, and Notch 1 [30,34].

In contrast, clear-cell EOC and endometrioid EOC share mutations in genes like ARID1A, PIK3CA, PTEN, and KRAS [35,36]. Mucinous EOCs frequently show KRAS mutations and HER2 amplifications [37,38], while low-grade serous EOC is characterized by activation of the mitogen-activated protein kinase (MAPK) pathway through mutations in NRAS, KRAS, or BRAF [39,40].

The expression of TOP2A in EOC varies depending on the studies, which, however, predominantly include high-grade serous histotypes. Van der Zee et al. [41] found TOP2A expression in 65% of patients (N = 54). Interestingly, the two cases of clear-cell EOC included in the study did not show TOP2A expression, while the single mucinous case had low TOP2A expression. In another study [42], nuclear immunoreactivity of TOP2A was observed in 94.7% of EOC cases (126 out of 133 patients), with most cases exhibiting moderate-to-strong intensity. Unfortunately, the authors did not specify the expression of TOP2A among histotypes different from the high-grade serous histotype. Ghisoni et al. [43] investigated TOP2A expression in 101 patients affected by EOC (79 serous): nuclear TOP2A expression was observed in all cases, with expression levels ranging from 2% to 48% of the nuclear cell population and an average of 18%.

**Figure 1:**
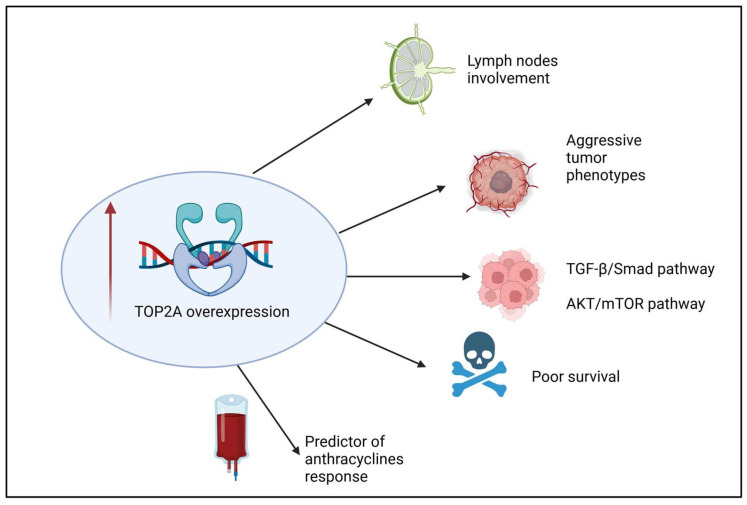
Relationship between TOP2A overexpression and epithelial ovarian cancer.

A comprehensive gene expression analysis of EOC samples from 62 patients (mostly of the high-grade serous histotype) identified a distinct pattern of TOP2A mRNA and protein levels in both tumor epithelial and adjacent stromal cells, which change in response to platinum-based chemotherapy. In untreated patients, tumor cells exhibited high levels of TOP2A expression, which was significantly reduced after platinum treatment. Conversely, the stromal cells in recurrent EOC showed higher levels of TOP2A protein compared to those in primary tumors. This differential expression suggests that stromal cells might play a role in the development of chemotherapy resistance in EOC [20]. Increased TOP2A expression is linked to higher-grade and advanced-stage tumors [44]. Furthermore, high expression correlates with poorer survival, and a significant association between nodal stage (pN) and both nuclear and cytoplasmic TOP2A expression was observed (*p* = 0.030 and *p* = 0.034, respectively) (Figure 1). In a cell culture study [30], TOP2A expression was significantly elevated in high-grade serous EOC cell lines (SKOV3 and HEY). The knockdown of TOP2A in these cell lines led to a marked reduction in their proliferation, migration, and invasion capabilities. Additionally, phosphorylated Smad2 and Smad3 levels, crucial components of the TGF-β/Smad pathway that regulate tumorigenesis, significantly decreased following TOP2A knockdown. Another study identified that Rhophilin Rho GTPase binding protein 1 antisense RNA1 (RHPN1-AS1), miR-6884-5p, and TOP2A were expressed in OC tissues or cell lines. RHPN1-AS1 has been shown to facilitate EOC development by acting as a sponge for miR-6884-5p, thereby freeing TOP2A [45]. Recently, it has been observed that TOP2A can control OC cell growth via AKT/mTOR pathway modulation [46] (Figure 1).

## 5. Predictive Role of TOP2A

TOP2A overexpression in EOC is associated with progression and recurrence and predicts poor survival outcomes [44,47]. A study [48] explored the role of TOP2A gene copy gain in predicting EOC’s response to PLD in 38 patients. Patients’ derived xenografts of OC were used to assess the correlation between TOP2A protein expression and response to PLD. By examining the expression levels, genetic alterations, and enzyme activity of TOP2A, it was found that an increase in TOP2A gene copies is associated with a better therapeutic response to PLD in patients with relapsed EOC. The findings suggest that TOP2A could serve as a valuable biomarker to identify patients who are more likely to benefit from PLD treatment, thus helping in the personalization of therapeutic strategies for ovarian cancer. This study underscores the importance of genetic profiling in improving treatment outcomes and highlights the potential of TOP2A as a predictor of response to PLD in ovarian cancer.

Another retrospective study on 101 patients with EOC highlighted the potential role of TOP2A as a marker of response to PLD (Figure 1). The authors suggest that a TOP2A expression value above 18% is associated with a higher probability of response to PLD and a longer PFS if the patients are treated with PLD monotherapy [43]. Other authors considered the expression of TOP2A in a panel of six hub genes as a predictive and prognostic marker [49].

## 6. Drugs That Target TOP2A

The main class of chemotherapeutic agents targeting TOP2A is anthracyclines. These products were first extracted from bacterial Streptomyces species and have antibiotic and antitumor activity. The drug class of anthracyclines includes doxorubicin (DOX), valrubicin, daunorubicin, epirubicin, and idarubicin. DOX is used in the treatment of breast cancer, various types of leukemia, lymphomas, and sarcomas, as well as EOC [19]. Etoposide is another class of drug that targets TOPA2. It is a semisynthetic glycosidic derivative of podophyllotoxin, derived from the rhizome of Podophyllum peltatum and other plants of the genus Podophyllum [50].

Drugs that target topoisomerases stabilize the cleavage complex, resulting in the accumulation of fatal DNA double-strand breaks. However, cancer cells can develop resistance through mechanisms such as single-point mutations in TOP2, alterations in gene expression, or the regulation of post-translational modifications (PTMs). Aberrant PTMs are frequently observed in cancer cells and are a distinguishing characteristic. Among these, phosphorylation is the most common PTM. Different studies have shown that the status of hyperphosphorylation is responsible for resistance to chemotherapy [51]. Matsumoto et al. observed that hyperphosphorylation is linked to drug resistance in breast cancer cells [52].

**Figure 2:**
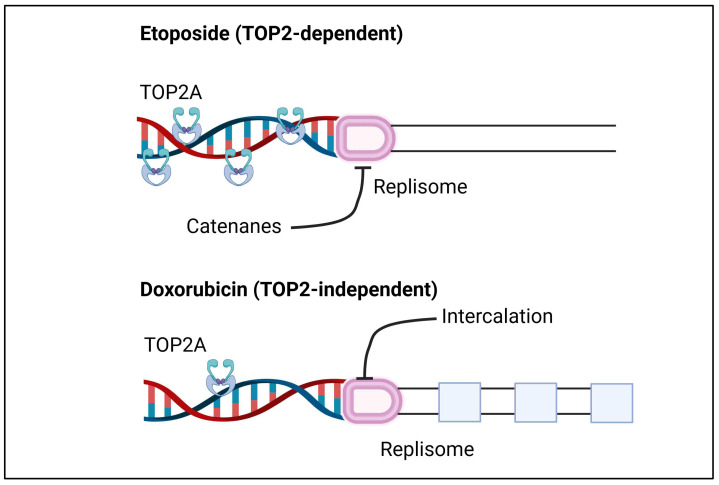
Different interactions of etoposide and doxorubicin with TOP2A. Figure adapted from the paper by Van Ravenstein et al. [54].

Etoposide and DOX trap Top2cc, leading to persistent DNA double-strand breaks which can induce programmed cell death in cancer cells. The effectiveness of TOP2 target drugs relies on the expression and nuclear localization of the enzyme. Cells that lack TOP2A expression or nuclear localization are relatively resistant to these drugs. Certain post-translational modifications have identified alternative splice variants of the TOP2A protein that do not localize to the nucleus, making cells expressing these variants resistant to TOP2A poisons [53]. Etoposide and DOX disrupt DNA replication via distinct pathways [54]. Etoposide causes TOP2-dependent DNA breaks and stalls replication forks by trapping TOP2 behind them. Conversely, DOX does not cause significant DNA breakage; it intercalates into the parental DNA, stalling replication forks in a TOP2-independent manner. In human cells, etoposide stalls replication forks through a TOP2-dependent mechanism, while doxorubicin does so independently of TOP2A (Figure 2). Despite these differences, both drugs exhibit TOP2-dependent cytotoxicity. Therefore, although etoposide and DOX inhibit DNA replication through different mechanisms, they share common genetic requirements for their cytotoxic effects [54].

Elton et al. reviewed the TOP2A splice variants and examined the role of alternative splicing as a cause of drug resistance, which is predominant in some cancer cell lines [55].

DOX and other anthracyclines have some negative effects, in particular cardiotoxicity, which can elicit congestive heart failure and dilative cardiomyopathy. These negative effects usually arise within a year, but very late forms of cardiac dysfunction have been described. The cardiomyopathy induced by anthracycline has some particular ultrastructural features that have been revealed during patients’ endomyocardial biopsies, including the loss of myofibrils, dilation of the sarcoplasmic reticulum, cytoplasmic vacuolization, swelling of mitochondria, and an increased number of lysosomes [13,56,57].

In cases of OC recurrence, anthracyclines can be used as single-agent drugs for patients who are refractory or resistant to platinum. They can be combined with trabectedin for those who are partially sensitive to platinum, and with platinum for those who are platinum-sensitive. PLD was introduced for recurrent OC, following a randomized trial that demonstrated its superiority over topotecan in a platinum-resistant OC setting, with a PFS of 108 weeks compared to 71.1 weeks (*p* = 0.008) [58]. In the CALYPSO trial [59], focusing on platinum-sensitive EOC, the combination of carboplatin and PLD showed improved progression-free survival (PFS) compared to the standard carboplatin and paclitaxel regimen (11.3 months versus 9.4 months; HR: 0.821, *p* = 0.005). For partially platinum-sensitive patients, the HR for PFS was 0.73 (CI: 0.58–0.90; *p* = 0.004), with median PFSs of 9.4 months for carboplatin and PLD and 8.8 months for carboplatin and paclitaxel. A recent analysis of patients with a treatment-free interval greater than 24 months found similar efficacy between the two regimens for PFS. However, carboplatin and PLD were recommended as the preferred treatment due to their favorable risk–benefit profile [60]. A systematic review and meta-analysis of 14 randomized trials, encompassing a total of 5760 patients, investigated the efficacy of PLD in the treatment of EOC [61]. The primary objective was to compare PLD-based treatments with other therapeutic regimens, focusing on OS, PFS, response rate, CA125 response, and toxicity profiles. Key findings indicated that there is no significant difference in OS between PLD-based treatments and other regimens. However, a significant improvement in PFS was observed with PLD-based treatments, particularly in second-line and platinum-sensitive subgroups [62].

The response rate and CA125 responses were similar between PLD-based treatments and other regimens. The toxicity profiles were also comparable, with no major differences in common hematological toxicities.

In summary, while PLD-based regimens do not improve OS, they offer a marginal benefit in PFS, especially for certain subgroups of patients, without significantly increasing toxicity [62].

Etoposide can be considered a treatment in recurrent platinum-refractory EOCs, but its efficacy in survival is modest [63].

Most of the studies that considered the use of drugs that target TOP2A have predominantly included high-grade serous tumors. Non-serous tumors are typically chemo-resistant [64,65,66]; however, resistance to doxorubicin is less commonly observed in mucinous and endometrioid carcinomas [13,65]. This indicates that these histologic types may benefit from including anthracyclines in their treatment protocols.

## 7. Advances in Targeting TOP2A in Ovarian Cancer

For this section, we conducted a literature review of studies published between 2010 and 2024, using databases such as Medline, PubMed, Scopus, Cochrane Library, and Web of Science. Key Medical Subject Heading (MeSH) search terms used included TOP2A, topoisomerase 2A AND/OR anthracyclines, etoposide, DOX, and pegylated DOX. We considered only studies that introduced new formulations or methods of administration, as well as studies that combined DOX with drugs other than the traditional doublet with carboplatin.

A summary of the published data from exploring new strategies with TOP2A inhibitors is available in Table 1.

### 7.1. New Formulations

Recent advancements in the formulation of drugs that target TOP2A have led to the development of novel delivery systems that enhance the drug’s pharmacokinetic properties. These new formulations aim to improve the therapeutic efficacy of doxorubicin while minimizing its associated toxicities. By optimizing the delivery and distribution of the drug, these innovations seek to provide better outcomes for patients undergoing treatment for various malignancies.

A novel approach uses dual-targeted nanoparticles responsive to glutathione (GSH) to deliver DOX and interfering RNAs (siRNAs) targeting surviving Bcl-2 and ABCG2 to OC stem cells [86]. These nanoparticles, formed by electrostatically assembling anionic siRNAs and cationic-disulfide-bond crosslinking-branched polyethyleneimine (SSBPEI)–DOX, release siRNA and DOX in OC stem cells due to selective reduction in the disulfide bonds in the tumor microenvironment. This siRNA cocktail specifically silences three critical genes, while DOX induces apoptosis or necrosis. iRGD peptides and a “sheddable” hyaluronic acid coating enhance targeting by binding to neuropilin-1 (NRP1) and CD44 [86]. The multifunctional delivery system shows excellent biocompatibility, precise targeting, and potent anti-OC effects, suggesting its potential to prevent OC metastasis and relapse.

Another study examined betulinic acid, a natural compound, and the chemical drug lonidamine as chemosensitizers in combination with DOX for EOC treatment. The researchers developed pH-sensitive peptide derivatives to create a multifunctional drug delivery system, targeting pH levels to deliver both DOX and betulinic acid (or lonidamine). The combination of DOX and betulinic acid exhibited superior therapeutic effects and reduced cardiotoxicity compared to the DOX and lonidamine combination in SKVO3 cells. Using micellar systems with DOX and betulinic acid enabled a burst release at the tumor site, improving antitumor efficacy and minimizing off-target effects. Conversely, micelles with DOX and lonidamine had fewer negative impacts on cardiac function and leukocyte counts in SKVO3 subcutaneous xenograft models [87].

A pH-sensitive liposome formulation (pHSL) co-loaded with tariquidar (TQR) and DOX was developed to address multidrug resistance. This formulation exhibited high stability at pH 7.4 and exceptional sensitivity in acidic environments, enhancing the delivery of TQR and DOX into cells. Cellular experiments demonstrated that pHSL/TQR/DOX 0.05 could nearly restore drug sensitivity in OVCAR8/ADR cells [88].

Other researchers have achieved similar results with transferrin- and octa arginine-modified liposomes loaded with DOX, which showed high cytoplasmic accumulation of DOX in OC cells, facilitating nuclear delivery and internalization via receptor-mediated endocytosis and macropinocytosis [89]. Two phase I trials using DOX hydrochloride liposomes are currently ongoing (NCT03681548, NCT02237690) [90].

Polymeric micelles incorporating DOX and rhein (RHE) (nano-DOX/RHE) were created to tackle drug resistance in OC cells and boost the therapeutic efficiency of DOX. Various parameters such as morphology, particle size (around 25 nm), zeta potential, release profile in vitro, cell proliferation, and cytotoxicity were assessed. The study found that DOX and RHE could be effectively encapsulated in micelle nanoparticles, and in vitro, experiments showed a sustained release of these drugs into DOX-resistant SKOV3 cells (SKOV3/DOX). Nano-DOX/RHE displayed enhanced cytotoxicity and significant apoptosis-inducing effects in SKOV3/DOX cells. Additionally, nano-DOX/RHE showed improved cancer-targeting capability, increasing antitumor efficacy with minimal toxicity [91].

### 7.2. Pressurized Intraperitoneal Aerosol Chemotherapy

An early study evaluated the efficacy and safety of Pressurized Intraperitoneal Aerosol Chemotherapy (PIPAC) using cisplatin and DOX in women with recurrent, platinum-resistant EOC. Conducted as a preliminary clinical trial, the research included patients who had previously undergone extensive treatments. The results demonstrated that PIPAC could be administered safely with manageable toxicity levels. The treatment showed promising activity, with a notable proportion of patients achieving disease control [67].

The same study group evaluated 99 women who underwent 252 PIPAC procedures. The primary outcomes measured were objective tumor response (OTR), peritoneal carcinomatosis index (PCI) improvement, and ascites volume reduction. The results showed an OTR of 76% among patients who underwent more than one PIPAC procedure, with significant decreases in ascites volume and improved PCI. Quality-of-life metrics also improved during therapy [68]. Subsequently, a retrospective study aimed to determine the dose-limiting toxicity and maximum tolerated dose of intraperitoneal (IP) cisplatin and DOX administered as PIPAC in patients with recurrent EOC and peritoneal carcinomatosis. The results indicated that PIPAC with cisplatin and DOX was generally well tolerated, with manageable toxicities [69]. Another study compared the concentrations of cisplatin and DOX in ascites and peritoneal tissue before and after the administration of PIPAC. The concentrations of these chemotherapeutic agents were measured using gas chromatography, finding that PIPAC results in substantial chemotherapy absorption in both ascites and peritoneal tissues, indicating a dual cytotoxic effect through direct uptake into peritoneal tumor nodules and via ascites. Repeated PIPAC treatments lead to the accumulation of DOX in the peritoneum, suggesting a cumulative cytotoxic impact of DOX with successive PIPAC applications [92].

More recently, a single-arm, phase II trial involved 43 women who underwent PIPAC treatment with DOX and cisplatin. The clinical benefit rate (CBR) was 82%, with 45% of patients completing three cycles of PIPAC. The median time from relapse to disease progression was 12 months, and the median OS was 27 months [70]. PIPAC with cisplatin and DOX appears to be a safe and potentially effective treatment option for patients with OC, showing encouraging trends in survival and manageable toxicity levels; however, further research is needed to confirm these findings and explore its combination with systemic chemotherapy [93]. A phase 2 trial [71] evaluated the efficacy of adding IP cisplatin and etoposide to standard first-line IV chemotherapy for patients with advanced EOC. The primary endpoint was the 12-month non-progression rate (NPR). The results showed that the IP/IV arm had a significantly higher 12-month NPR (81.9%) than the IV arm (64.2%), with a median PFS of 22.4 months versus 16.8 months, respectively. Although there were more grade 3/4 adverse events in the IP/IV arm, the treatment was considered acceptable. The study concluded that IP chemotherapy improved NPR and PFS before IV chemotherapy, warranting long-term follow-up to assess OS.

### 7.3. Trabectedin

Trabectedin operates through two main mechanisms. Firstly, it targets the DNA of cancer cells, leading to cell cycle arrest and apoptosis. Secondly, it affects the tumor microenvironment by modulating components such as tumor-associated macrophages and vascular endothelial cells. These combined actions hinder the tumor’s growth and its ability to evade the immune system, enhancing trabectedin’s overall antitumor efficacy [94].

A phase 3, randomized, open-label, multicenter trial assessed the safety and effectiveness of combining trabectedin and PLD therapy for recurrent EOC. Enrolled patients were randomized into two groups: trabectedin + PLD or only PLD [72]. The median OS was 23.8 months for the trabectedin + PLD group compared to 22.2 months for the PLD group (HR: 0.92, 95% CI: 0.73–1.18; *p* = 0.52). The median PFS was 7.52 months for trabectedin + PLD versus 7.26 months for PLD (HR: 0.93, 95% CI: 0.76–1.15; *p* = 0.52). The ORR was 46% for the trabectedin + PLD group, compared to 35.9% for the PLD group (OR: 1.52, 95% CI: 1.07–2.16; *p* = 0.01) [72].

For patients with BRCA1/2 mutations, the median OS was 34.2 months for trabectedin + PLD compared to 20.9 months for PLD (HR: 0.54, 95% CI: 0.33–0.90; *p* = 0.016). The median PFS was 10.1 months with trabectedin + PLD versus 7.6 months with PLD (HR: 0.72, 95% CI: 0.48–1.08; *p* = 0.039). Among patients with BRCA1/2 mutations and a PFI of 6–12 months, the median OS was 31.5 months for trabectedin + PLD compared to 14.9 months for PLD (HR: 0.37, 95% CI: 0.17–0.82; *p* = 0.011) [73].

Another phase III international randomized study (INOVATYON/ENGOT-ov5) compared trabectedin + PLD followed by platinum at progression versus carboplatin + PLD in patients with recurrent EOC. The study did not achieve the primary endpoint of OS. The carboplatin + PLD combination remains the standard for patients with recurrent EOC and a 6–12-month PFI. Trabectedin + PLD is effective for patients with persistent platinum toxicities [74].

A multicenter, retrospective analysis compared the efficacy of PLD + trabectedin in patients previously treated with PARPis (cases) versus PARPi-naïve patients (controls). The PFS was 11 months (95% CI: 10–12) in the control group compared to 8 months (95% CI: 6–9) in the case group (*p* = 0.0017). The CBR shows a progression HR of 2.55 (95% CI: 1.28–5.06) for the case group (*p* = 0.008). The study revealed a significant difference in PFS, suggesting that prior exposure to PARP inhibitors might reduce the efficacy of PLD + trabectedin [95].

### 7.4. Sodium Valproate Acid

Sodium valproate acid (VPA) is a short-chain fatty acid that functions as a histone deacetylase inhibitor and has demonstrated antitumor activity in preclinical models of platinum-resistant EOC, and it has been shown to work synergistically with conventional cytotoxic agents [75,96].

A phase II study explored the combination of VPA and oral etoposide in patients with platinum-resistant EOC. The treatment regimen consisted of oral VPA followed by oral etoposide. The results showed an ORR of 0% and a CBR of 11%. The median PFS was recorded at 2 months, and the median OS was 7 months.

Adding VPA to oral etoposide did not enhance response rates compared to historical data with single-agent etoposide [97].

### 7.5. PARPi

Data from an in vitro study showed a synergism of the combination of the PARPi olaparib plus doxorubicin in 2D and spheroid models of OC. The authors studying OC cell lines UWB1-289 (*BRCA*-mutated and wild-type genotypes) demonstrated the superiority of combination therapy over monotherapy, resulting in a significantly increased number of double-strand breaks [98].

The GEICO1601-ROLANDO trial was a single-arm phase II study demonstrating the significant activity of combining PLD with olaparib for treating platinum-resistant OC, regardless of BRCA status.

The study was divided into two treatment phases: during the induction phase, patients received PLD and olaparib tablets for up to six cycles. In the maintenance phase, patients continued with olaparib as monotherapy until disease progression or unacceptable toxicity developed.

With a median follow-up of 10.2 months, the 6-month PFS rate was 47%, and the median PFS was 5.8 months [76].

Another phase II study evaluated the efficacy and safety of an oral combination therapy of niraparib and etoposide in patients with platinum-resistant or refractory OC. Participants were administered niraparib along with oral etoposide. Niraparib was continued until disease progression or intolerable toxicity. The primary endpoint was PFS, with a median PFS of 4.2 months. The overall ORR was 26.9%, and the disease control rate (DCR) was 57.7%. Notably, patients with BRCA mutations and homologous recombination deficiency (HRD) showed higher response rates. The combination of niraparib and etoposide demonstrated antitumor activity and manageable safety in patients with platinum-resistant or refractory EOC, particularly in those with BRCA mutations or HRD [99].

### 7.6. Immune Checkpoint Inhibitors

Immune checkpoint inhibitors (ICIs), including anti-PD-1 and anti-PD-L1 antibodies, have shown promise in treating some gynecological cancers by blocking the PD-1/PD-L1 interaction and enhancing the immune response [100].

ICIs have proven effective in cervical cancer, particularly in advanced or recurrent cases, improving OS rates and providing new treatment options [101,102,103]. In endometrial cancer, ICIs have shown potential in reducing tumor size and slowing disease progression, especially in mismatch repair-deficient (dMMR) tumors [104,105,106]. Ongoing research is focused on optimizing ICI use and improving patient outcomes in these malignancies [107,108]. However, despite encouraging preclinical results for OC treatment, clinical studies have been largely disappointing, showing limited benefits [109,110,111,112,113]. In this scenario, some authors have studied the role of ICIs in addition to some TOP2A inhibitors in recurrent OC.

A phase II study evaluated the combination of pembrolizumab (anti PD-1) with PLD in patients with recurrent platinum-resistant EOC, fallopian tube cancer, or peritoneal cancer. The study enrolled patients who had previously undergone one or two lines of cytotoxic therapy for recurrent or persistent disease. Participants received pembrolizumab along with PLD. The primary endpoint was the CBR, which included complete responses (CRs), partial responses (PRs), and stable disease (SD) lasting at least 24 weeks. Results showed a CBR of 52.2%, with an ORR of 26.1%, including one complete response and five partial responses. The median PFS was 4.1 months, and the median OS was 13.1 months. The combination therapy was generally well tolerated, with manageable side effects [77,78].

The PemBOv trial investigated the combination of pembrolizumab and bevacizumab, with or without PLD-based chemotherapy, in patients with platinum-resistant OC.

The trial included three cohorts: pembrolizumab plus PLD, pembrolizumab plus bevacizumab, and pembrolizumab, bevacizumab, and PLD. The primary endpoints were safety and efficacy. The results indicated that the combination therapies were generally well tolerated, with manageable side effects. The addition of bevacizumab to pembrolizumab showed enhanced responses in platinum-resistant OC patients, suggesting the potential benefits of this combination [79].

A phase III, multicenter, randomized, open-label study evaluated the efficacy and safety of nivolumab (antiPD-1) compared to chemotherapy (gemcitabine or PLD) in patients with platinum-resistant EOC. The study found that nivolumab did not improve OS and resulted in worse PFS compared to gemcitabine or PLD in these patients [80].

The phase III JAVELIN Ovarian 200 trial evaluated the efficacy and safety of avelumab (anti PD-L1), combined with PLD, compared to PLD alone in patients with platinum-resistant or refractory EOC.

The results of this study indicated that avelumab neither alone nor in combination with PLD significantly improved PFS or OS compared to PLD alone. Despite these findings, exploratory biomarker analyses revealed that higher expression of CD8+ T-cell signatures and higher tumor mutational burden were associated with longer PFS and OS in the avelumab-alone arm. Additionally, the presence of certain genetic markers, such as the high-affinity FCGR2A allele, correlated with better outcomes [81].

### 7.7. AVB-500

AVB-500 is a recombinant fusion protein dimer comprising a modified portion of the extracellular region of human AXL fused with a human IgG1 heavy chain. This protein has the potential to inhibit downstream signaling involved in various cancer-related processes, such as epithelial-to-mesenchymal transition, migration, invasion, angiogenesis, and immune suppression and evasion [114].

In a phase 1b study, patients received AVB-500 at three different dosages in combination with either paclitaxel (n = 23) or PLD (n = 30). The study reported no dose-limiting toxicities, and serum GAS6 levels were fully suppressed at all three dose levels of AVB-500. The combination of AVB-500 and paclitaxel demonstrated superior clinical activity compared to AVB-500 and PLD, with an ORR of 34.8% (8 out of 23 patients, including 2 complete responses) and median durations of response, PFS, and OS of 7.0, 3.1, and 10.3 months, respectively.

Subgroup analyses revealed that patients treated with AVB-500 and paclitaxel who had not received prior bevacizumab treatment or had AVB-500 trough levels above 13.8 mg/L experienced the best clinical responses. For these patients, the ORR, median PFS, and OS were at least 50%, 7.5 months, and 19 months, respectively [82].

### 7.8. Apatinib

The APPROVE trial [83] investigated the efficacy of combining apatinib, a VEGF receptor 2 tyrosine kinase inhibitor, with PLD in treating platinum-resistant recurrent OC. The study included 152 patients who were randomized to receive either the combination treatment or PLD alone. The median PFS was 5.8 months for the combination group compared to 3.3 months for the PLD-alone group. The combination treatment reduced the risk of disease progression or death by 56% compared to PLD alone.

The median OS was 23.0 months for the combination group versus 14.4 months for the PLD-alone group, although this difference was not statistically significant. Overall, the study suggests that apatinib plus PLD may be a promising alternative treatment for patients with platinum-resistant recurrent OC [83]. A phase 2, single-arm, prospective study [84] evaluated the efficacy and safety of combining apatinib with oral etoposide in platinum-resistant or platinum-refractory OC patients. The study included patients who received apatinib and oral etoposide. In the intention-to-treat population, 19 out of 35 patients (54%; 95% CI 36.6–71.2) achieved objective responses. The results showed promising efficacy with manageable toxicities, suggesting that this combination therapy could be a potential treatment option for these patients. A retrospective study [85] investigating the same combination on 33 recurrent platinum-resistant EOC patients reported no cases of complete response but an ORR and DCR of 36.4% and 78.8%, respectively. The median PFS was 5.6 months (95% CI, 4.1~7.1), and the median OS was 10.3 months (95% CI, 9.4~11.2). Of note, 18 patients (54.5%) discontinued the treatment.

## 8. Strengths and Limitations

This review provides a comprehensive overview of the current understanding of TOP2A’s role in ovarian cancer, highlighting its potential as a therapeutic target. We synthesized a wide range of studies, offering a nuanced understanding of TOP2A expression, genetic alterations, and enzyme activity in the context of OC. The narrative approach allows for a detailed exploration of the mechanistic insights and therapeutic implications of targeting TOP2A, which may not be fully captured in more systematic reviews. However, as a narrative review, this paper may lack the systematic rigor and quantitative synthesis typical of meta-analyses.

## 9. Conclusions and Future Directions

Targeting TOP2A is still an important strategy for OC, given its critical role in tumor cell proliferation and survival. The overexpression of TOP2A in OC correlates with poor prognosis and aggressive disease phenotypes, underscoring its potential as both a prognostic and predictive biomarker and a therapeutic target. Mechanistic studies have elucidated that TOP2A modulates key signaling pathways, which are pivotal in cancer cell growth and survival. Preclinical models and early clinical trials have demonstrated the efficacy of TOP2A inhibitors, either as monotherapy or in combination with other chemotherapeutic agents, in reducing tumor burden and improving patient outcomes, especially in platinum-sensitive recurrent disease. It should be noted, however, that most of the data derive from studies conducted on high-grade serous EOCs.

Recent clinical trials and studies have explored various therapies targeting TOP2A to improve OS and PFS in patients with EOC. For instance, the combination of trabectedin and PLD showed promising results in patients with BRCA1/2 mutations, significantly extending OS and PFS compared to PLD alone. Similarly, a study on apatinib combined with PLD demonstrated a notable increase in PFS for patients with platinum-resistant recurrent OC. In contrast, other treatments such as immunotherapy combined with DOX did not give the expected results. However, the efficacy of these combinations may vary, and further research is needed to validate these findings and optimize treatment strategies for improved patient outcomes.

Future research should focus on optimizing TOP2A-targeted therapies, exploring new formulations, and new combination regimens, and identifying biomarkers for patient stratification to enhance the clinical management of EOC.

## Figures and Tables

**Table 1:** Main results of studies exploring new strategies with TOP2A inhibitors.

Ref	Drug	Study Design	Clinical Setting	Primary Endpoints	Number of Patients Evaluated	OS	PFS	Other
Tempfer et al. [67]	Cisplatin + DOX (PIPAC)	Prospective	Recurrent platinum-resistant OC	OTR	18	/	/	6/34 OTR
Tempfer et al. [68]	Cisplatin + DOX (PIPAC)	Retrospective	Peritoneal carcinomatosis	OTR	99 (84 OC)	/	/	38/50 (76%) OTR (in women with more than one PIPAC procedure)
Tempfer et al. [69]	Cisplatin + DOX (PIPAC)	Phase I, single-arm, non-randomized, open-label	Recurrent OC	Maximum-tolerable dose	15	/	/	No dose-limiting toxicities were found
Vizzielli et al. [70]	Cisplatin + DOX (PIPAC)	Monocentric, single-arm, phase II trial	Recurrent platinum-resistant OC	CBR	43	Mean OS 27 months	12 months	CBR: 82%
Shi et al. [71]	Cisplatin + etoposide IP/EV arm vs. EV alone arm	Randomized, phase II trial	Stage III-IC EOC patients who underwent optimal debulking surgery	NPR	218		IP/EV vs. EV 22.4 vs. 16.8 months	
Monk et al. [72]	Trabectedin + PDL vs. PDL	Phase III, randomized, open-label, multicenter trial	Recurrent platinum-sensitive OC	OS	675	23.8 for trabectedin + DOX vs. 22.2 for DOX (months)	7.52 for trabectedin + DOX vs. 7.26 for DOX months	
Colombo et al. [73]	Trabectedin + PDL vs. carboplatin + PDL	Randomized phase III international study	Recurrent OC progressing within 6-12 months after last platinum line	OS	617	Not reached	/	
Turinetto et al. [74]	Trabectedin + PDL after exposure to PARPis or not	Multicenter, retrospective	Recurrent platinum-sensitive OC	PFS, CBR	135	/	11 months (control group) vs. 8 months (PARPi group)	CBR: HR for progression of 2.55 (1.28–5.06) for the PARPi group
Nakka et al. [75]	VPA + etoposide	Prospective, single-arm, open-label, phase II study,	Recurrent platinum-resistant OC	ORR	27	7 months	2 months	ORR: 0%
Zhou et al. [76]	Niraparib + etoposide	Multicenter single-arm phase II clinical trial	Platinum-resistant/refractory OC	PFS	29	/	4.2 months	ORR: 26.9%
Matulonis et al. [77]; Leet et al. [78]	Pembrolizumab + PLD	Phase II study	Recurrent platinum-resistant OC	CBR	26	13.1 months	4.1 months	CBR: 52.2% ORR: 26.1%
Michels et al. [79]	Pembrolizumab + bevacizumab vs. pembrolizumab + PLD vs. pembrolizumab + PLD + bevacizumab	Phase I trial	Platinum-resistant OC	Safety and efficacy				
Hamanishi et al. [80]	Nivolumab vs. gemcitabine or PLD	Phase III, multicenter, randomized, open-label trial	Platinum-resistant OC	OS	316	10.1 months for nivolumab, 12.1 for gemcitabine or PLD	2 months for nivolumab, 3.8 for gemcitabine or PLD	ORR: 7.6 for nivolumab, 13.2 for gemcitabine or PLD
Pujade-Lauraine et al. [81]	Avelumab vs. Avelumab + PLD vs. PLD	Open-label, parallel-group, three-arm, randomized, phase III trial	Platinum-resistant or refractory OC	OS, PFS	566	5.7 months for combination group, 13.1 months for PLD group, 11.8 months for avelumab group	3.7 months for combination group, 3.5 months for PLD group, and 1.9 months for avelumab group	
Fuh et al. [82]	AVB-500 + PLD	Phase Ib study	Platinum-resistant OC	/	30	4.2 months	3.6 months	ORR: 10.7%
Wang et al. [83]	Apatinib + PLD	Open-label, randomized, multicenter clinical trial	Platinum-resistant or refractory OC	PFS	152	23.0 months for apatinib + PLD vs. 14.4 months for PLD	5.8 months for apatinib + PLD vs. 3.3 months for PLD; *p* < 0.001	
Lan et al. [84]	Apatinib + etoposide	Phase II, single-arm, prospective study	Platinum-resistant or refractory OC	ORR	35	/	/	ORR: 54%
Huang et al. [85]	Apatinib + etoposide	Retrospective study	Recurrent platinum-resistant OC	ORR	33	10.3 months	5.6 months	ORR 36.4% DCR 78.8%

CBR: clinical benefit rate; DCR: disease control rate; DOX: doxorubicin; NPR: non-progression rate; OC: ovarian cancer; ORR: overall response rate; OS: overall survival; OTR: objective tumor response; PARPIs: Poly (ADP-ribose) polymerase inhibitors; PFS: progression-free survival; PIPAC: pressurized intraperitoneal aerosol chemotherapy; PLD: pegylated liposomal doxorubicin; VPA: sodium valproic acid.

## References

[1] Bray F., Laversanne M., Sung H., Ferlay J., Siegel R.L., Soerjomataram I., Jemal A. (2024). Global Cancer Statistics 2022: GLOBOCAN Estimates of Incidence and Mortality Worldwide for 36 Cancers in 185 Countries. CA Cancer J. Clin..

[2] Lheureux S., Gourley C., Vergote I., Oza A.M. (2019). Epithelial Ovarian Cancer. Lancet.

[3] Burger R.A., Brady M.F., Bookman M.A., Fleming G.F., Monk B.J., Huang H., Mannel R.S., Homesley H.D., Fowler J., Greer B.E. (2011). Incorporation of Bevacizumab in the Primary Treatment of Ovarian Cancer. N. Engl. J. Med..

[4] Perren T.J., Swart A.M., Pfisterer J., Ledermann J.A., Pujade-Lauraine E., Kristensen G., Carey M.S., Beale P., Cervantes A., Kurzeder C. (2011). A Phase 3 Trial of Bevacizumab in Ovarian Cancer. N. Engl. J. Med..

[5] Ledermann J.A., Raja F.A., Fotopoulou C., Gonzalez-Martin A., Colombo N., Sessa C. (2013). Newly Diagnosed and Relapsed Epithelial Ovarian Carcinoma: ESMO Clinical Practice Guidelines for Diagnosis, Treatment and Follow-Up. Ann. Oncol..

[6] DiSilvestro P., Colombo N., Harter P., González-Martín A., Ray-Coquard I., Coleman R.L. (2021). Maintenance Treatment of Newly Diagnosed Advanced Ovarian Cancer: Time for a Paradigm Shift?. Cancers.

[7] Goh J.C.H., Gourley C., Tan D.S.P., Nogueira-Rodrigues A., Elghazaly H., Edy Pierre M., Giornelli G., Kim B.-G., Morales–Vasquez F., Tyulyandina A. (2022). Optimizing Treatment Selection and Sequencing Decisions for First-Line Maintenance Therapy of Newly Diagnosed Advanced Ovarian Cancer–International Considerations amongst Upper Middle- and High-Income Countries (UMIC and HIC). Gynecol. Oncol. Rep..

[8] Luo J., Ou S., Wei H., Qin X., Jiang Q. (2022). Comparative Efficacy and Safety of Poly (ADP-Ribose) Polymerase Inhibitors in Patients With Ovarian Cancer: A Systematic Review and Network Meta-Analysis. Front. Oncol..

[9] Bogani G., Coleman R.L., Vergote I., van Gorp T., Ray-Coquard I., Oaknin A., Matulonis U., O’Malley D., Raspagliesi F., Scambia G. (2024). Mirvetuximab Soravtansine-Gynx: First Antibody/Antigen-Drug Conjugate (ADC) in Advanced or Recurrent Ovarian Cancer. Int. J. Gynecol. Cancer.

[10] Monk B.J., Herzog T.J., Kaye S.B., Krasner C.N., Vermorken J.B., Muggia F.M., Pujade-Lauraine E., Park Y.C., Parekh T.V., Poveda A.M. (2012). Trabectedin plus Pegylated Liposomal Doxorubicin (PLD) versus PLD in Recurrent Ovarian Cancer: Overall Survival Analysis. Eur. J. Cancer.

[11] Edwards S.J., Barton S., Thurgar E., Trevor N. (2015). Topotecan, Pegylated Liposomal Doxorubicin Hydrochloride, Paclitaxel, Trabectedin and Gemcitabine for Advanced Recurrent or Refractory Ovarian Cancer: A Systematic Review and Economic Evaluation. Health Technol. Assess..

[12] Ferrandina G., Amadio G., Paris I., Distefano M., Palluzzi E., De Vincenzo R., Ricci C., Scambia G. (2017). Real-World Management of Trabectedin/Pegylated Liposomal Doxorubicin in Platinum-Sensitive Recurrent Ovarian Cancer Patients: A National Survey. Int. J. Gynecol. Cancer.

[13] Ferrero A., Borghese M., Restaino S., Puppo A., Vizzielli G., Biglia N. (2022). Predicting Response to Anthracyclines in Ovarian Cancer. Int. J. Environ. Res. Public Health.

[14] Riccio A.A., Schellenberg M.J., Williams R.S. (2020). Molecular Mechanisms of Topoisomerase 2 DNA–Protein Crosslink Resolution. Cell. Mol. Life Sci..

[15] Deweese J.E., Osheroff M.A., Osheroff N. (2009). DNA Topology and Topoisomerases: Teaching a “Knotty” Subject. Biochem. Mol. Biol. Educ..

[16] Bush N.G., Evans-Roberts K., Maxwell A. (2015). DNA Topoisomerases. EcoSal Plus.

[17] Liu L.-M., Xiong D.-D., Lin P., Yang H., Dang Y.-W., Chen G. (2018). DNA Topoisomerase 1 and 2A Function as Oncogenes in Liver Cancer and May Be Direct Targets of Nitidine Chloride. Int. J. Oncol..

[18] McClendon A.K., Osheroff N. (2007). DNA Topoisomerase II, Genotoxicity, and Cancer. Mutat. Res. Fundam. Mol. Mech. Mutagen..

[19] Hevener K., Verstak T.A., Lutat K.E., Riggsbee D.L., Mooney J.W. (2018). Recent Developments in Topoisomerase-Targeted Cancer Chemotherapy. Acta Pharm. Sin. B.

[20] Chekerov R., Klaman I., Zafrakas M., Könsgen D., Mustea A., Petschke B., Lichtenegger W., Sehouli J., Dahl E. (2006). Altered Expression Pattern of Topoisomerase IIalpha in Ovarian Tumor Epithelial and Stromal Cells after Platinum-Based Chemotherapy. Neoplasia.

[21] Nitiss J.L. (2009). Targeting DNA Topoisomerase II in Cancer Chemotherapy. Nat. Rev. Cancer.

[22] Romero A., Caldés T., Díaz-Rubio E., Martín M. (2012). Topoisomerase 2 Alpha: A Real Predictor of Anthracycline Efficacy?. Clin. Transl. Oncol..

[23] Chen T., Sun Y., Ji P., Kopetz S., Zhang W. (2015). Topoisomerase IIα in Chromosome Instability and Personalized Cancer Therapy. Oncogene.

[24] Uusküla-Reimand L., Wilson M.D. (2022). Untangling the Roles of TOP2A and TOP2B in Transcription and Cancer. Sci. Adv..

[25] Wong N., Yeo W., Wong W.-L., Wong N.L.-Y., Chan K.Y.-Y., Mo F.K.-F., Koh J., Chan S.L., Chan A.T.-C., Lai P.B.-S. (2009). TOP2A Overexpression in Hepatocellular Carcinoma Correlates with Early Age Onset, Shorter Patients Survival and Chemoresistance. Int. J. Cancer.

[26] Wang X., Wang J., Lyu L., Gao X., Cai Y., Tang B. (2022). Oncogenic Role and Potential Regulatory Mechanism of Topoisomerase IIα in a Pan-Cancer Analysis. Sci. Rep..

[27] An X., Xu F., Luo R., Zheng Q., Lu J., Yang Y., Qin T., Yuan Z., Shi Y., Jiang W. (2018). The Prognostic Significance of Topoisomerase II Alpha Protein in Early Stage Luminal Breast Cancer. BMC Cancer.

[28] Depowski P.L., Rosenthal S.I., Brien T.P., Stylos S., Johnson R.L., Ross J.S. (2000). Topoisomerase IIα Expression in Breast Cancer: Correlation with Outcome Variables. Mod. Pathol..

[29] Yeo C.Q.X., Alexander I., Lin Z., Lim S., Aning O.A., Kumar R., Sangthongpitag K., Pendharkar V., Ho V.H.B., Cheok C.F. (2016). p53 Maintains Genomic Stability by Preventing Interference between Transcription and Replication. Cell Rep..

[30] The Cancer Genome Atlas Research Network (2011). Integrated Genomic Analyses of Ovarian Carcinoma. Nature.

[31] Kroeger P.T., Drapkin R. (2017). Pathogenesis and Heterogeneity of Ovarian Cancer. Curr. Opin. Obstet. Gynecol..

[32] Lheureux S., Braunstein M., Oza A.M. (2019). Epithelial Ovarian Cancer: Evolution of Management in the Era of Precision Medicine. CA Cancer J. Clin..

[33] Pesenti C., Beltrame L., Velle A., Fruscio R., Jaconi M., Borella F., Cribiù F.M., Calura E., Venturini L.V., Lenoci D. (2022). Copy Number Alterations in Stage I Epithelial Ovarian Cancer Highlight Three Genomic Patterns Associated with Prognosis. Eur. J. Cancer.

[34] D’Ambrosio C., Erriquez J., Arigoni M., Capellero S., Mittica G., Ghisoni E., Borella F., Katsaros D., Privitera S., Ribotta M. (2020). PIK3R1W624R Is an Actionable Mutation in High Grade Serous Ovarian Carcinoma. Cells.

[35] Mc Conechy M.K., Ding J., Senz J., Yang W., Melnyk N., Tone A.A., Prentice L.M., Wiegand K.C., McAlpine J.N., Shah S.P. (2014). Ovarian and Endometrial Endometrioid Carcinomas Have Distinct CTNNB1 and PTEN Mutation Profiles. Mod. Pathol..

[36] Itamochi H., Oishi T., Oumi N., Takeuchi S., Yoshihara K., Mikami M., Yaegashi N., Terao Y., Takehara K., Ushijima K. (2017). Whole-Genome Sequencing Revealed Novel Prognostic Biomarkers and Promising Targets for Therapy of Ovarian Clear Cell Carcinoma. Br. J. Cancer.

[37] Ledermann J.A., Luvero D., Shafer A., O’Connor D., Mangili G., Friedlander M., Pfisterer J., Mirza M.R., Kim J.-W., Alexandre J. (2014). Gynecologic Cancer InterGroup (GCIG) Consensus Review for Mucinous Ovarian Carcinoma. Int. J. Gynecol. Cancer.

[38] Borella F., Mitidieri M., Cosma S., Benedetto C., Bertero L., Fucina S., Ray-Coquard I., Carapezzi A., Ferraioli D. (2023). Update on Prognostic and Predictive Markers in Mucinous Ovarian Cancer. Cancers.

[39] Slomovitz B., Gourley C., Carey M.S., Malpica A., Shih I.-M., Huntsman D., Fader A.N., Grisham R.N., Schlumbrecht M., Sun C.C. (2020). Low-Grade Serous Ovarian Cancer: State of the Science. Gynecol. Oncol..

[40] Manning-Geist B., Gordhandas S., Liu Y.L., Zhou Q., Iasonos A., Da Cruz Paula A., Mandelker D., Long Roche K., Zivanovic O., Maio A. (2022). MAPK Pathway Genetic Alterations Are Associated with Prolonged Overall Survival in Low-Grade Serous Ovarian Carcinoma. Clin. Cancer Res..

[41] van der Zee A.G., de Vries E.G., Hollema H., Kaye S.B., Brown R., Keith W.N. (1994). Molecular analysis of the topoisomerase II alpha gene and its expression in human ovarian cancer. Ann. Oncol..

[42] Faggad A., Darb-Esfahani S., Wirtz R., Sinn B., Sehouli J., Könsgen D., Lage H., Weichert W., Noske A., Budczies J. (2009). Topoisomerase IIalpha mRNA and Protein Expression in Ovarian Carcinoma: Correlation with Clinicopathological Factors and Prognosis. Mod. Pathol..

[43] Ghisoni E., Maggiorotto F., Borella F., Mittica G., Genta S., Giannone G., Katsaros D., Sciarrillo A., Ferrero A., Sarotto I. (2019). TOP2A as Marker of Response to Pegylated Lyposomal Doxorubicin (PLD) in Epithelial Ovarian Cancers. J. Ovarian Res..

[44] Gao Y., Zhao H., Ren M., Chen Q., Li J., Li Z., Yin C., Yue W. (2020). TOP2A Promotes Tumorigenesis of High-Grade Serous Ovarian Cancer by Regulating the TGF-β/Smad Pathway. J. Cancer.

[45] Cui S., Li F. (2021). RHPN1-AS1 Promotes Ovarian Carcinogenesis by Sponging miR-6884-5p Thus Releasing TOP2A mRNA. Oncol. Rep..

[46] Zhang K., Zheng X., Sun Y., Feng X., Wu X., Liu W., Gao C., Yan Y., Tian W., Wang Y. (2024). TOP2A Modulates Signaling via the AKT/mTOR Pathway to Promote Ovarian Cancer Cell Proliferation. Cancer Biol. Ther..

[47] Kucukgoz Gulec U., Gumurdulu D., Guzel A.B., Paydas S., Seydaoglu G., Acikalin A., Khatib G., Zeren H., Vardar M.A., Altintas A. (2014). Prognostic Importance of Survivin, Ki-67, and Topoisomerase IIα in Ovarian Carcinoma. Arch. Gynecol. Obstet..

[48] Erriquez J., Becco P., Olivero M., Ponzone R., Maggiorotto F., Ferrero A., Scalzo M.S., Canuto E.M., Sapino A., Verdun di Cantogno L. (2015). TOP2A gene copy gain predicts response of epithelial ovarian cancers to pegylated liposomal doxorubicin: TOP2A as marker of response to PLD in ovarian cancer. Gynecol. Oncol..

[49] Shen J., Yu S., Sun X., Yin M., Fei J., Zhou J. (2019). Identification of Key Biomarkers Associated with Development and Prognosis in Patients with Ovarian Carcinoma: Evidence from Bioinformatic Analysis. J. Ovarian Res..

[50] Montecucco A., Zanetta F., Biamonti G. (2015). Molecular Mechanisms of Etoposide. EXCLI J..

[51] Lotz C., Lamour V. (2020). The Interplay between DNA Topoisomerase 2α Post-Translational Modifications and Drug Resistance. Cancer Drug Resist..

[52] Matsumoto Y., Takano H., Fojo T. (1997). Cellular Adaptation to Drug Exposure: Evolution of the Drug-Resistant Phenotype. Cancer Res..

[53] Gmeiner W.H., van Waardenburg R.C.A.M. (2021). Targeting DNA Topoisomerases: Past & Future. Cancer Drug Resist..

[54] Van Ravenstein S.X., Mehta K.P., Kavlashvili T., Byl J.A.W., Zhao R., Osheroff N., Cortez D., Dewar J.M. (2022). Topoisomerase II poisons inhibit vertebrate DNA replication through distinct mechanisms. EMBO J..

[55] Elton T.S., Ozer H.G., Yalowich J.C. (2020). Effects of DNA Topoisomerase IIα Splice Variants on Acquired Drug Resistance. Cancer Drug Resist..

[56] Menna P., Salvatorelli E., Minotti G. (2008). Cardiotoxicity of Antitumor Drugs. Chem. Res. Toxicol..

[57] Minotti G., Menna P., Salvatorelli E., Cairo G., Gianni L. (2004). Anthracyclines: Molecular Advances and Pharmacologic Developments in Antitumor Activity and Cardiotoxicity. Pharmacol. Rev..

[58] Gordon A.N., Fleagle J.T., Guthrie D., Parkin D.E., Gore M.E., Lacave A.J. (2001). Recurrent Epithelial Ovarian Carcinoma: A Randomized Phase III Study of Pegylated Liposomal Doxorubicin Versus Topotecan. J. Clin. Oncol..

[59] Pujade-Lauraine E., Wagner U., Aavall-Lundqvist E., Gebski V., Heywood M., Vasey P.A., Volgger B., Vergote I., Pignata S., Ferrero A. (2010). Pegylated Liposomal Doxorubicin and Carboplatin Compared With Paclitaxel and Carboplatin for Patients With Platinum-Sensitive Ovarian Cancer in Late Relapse. J. Clin. Oncol..

[60] Gladieff L., Ferrero A., De Rauglaudre G., Brown C., Vasey P., Reinthaller A., Pujade-Lauraine E., Reed N., Lorusso D., Siena S. (2012). Carboplatin and Pegylated Liposomal Doxorubicin versus Carboplatin and Paclitaxel in Partially Platinum-Sensitive Ovarian Cancer Patients: Results from a Subset Analysis of the CALYPSO Phase III Trial. Ann. Oncol..

[61] Mahner S., Meier W., Du Bois A., Brown C., Lorusso D., Dell’Anna T., Cretin J., Havsteen H., Bessette P., Zeimet A.G. (2015). Carboplatin and Pegylated Liposomal Doxorubicin versus Carboplatin and Paclitaxel in Very Platinum-Sensitive Ovarian Cancer Patients: Results from a Subset Analysis of the CALYPSO Phase III Trial. Eur. J. Cancer.

[62] Staropoli N., Ciliberto D., Botta C., Fiorillo L., Grimaldi A., Lama S., Caraglia M., Salvino A., Tassone P., Tagliaferri P. (2014). Pegylated Liposomal Doxorubicin in the Management of Ovarian Cancer: A Systematic Review and Metaanalysis of Randomized Trials. Cancer Biol. Ther..

[63] Baert T., Ferrero A., Sehouli J., O’Donnell D.M., González-Martín A., Joly F., van der Velden J., Blecharz P., Tan D.S.P., Querleu D. (2021). The Systemic Treatment of Recurrent Ovarian Cancer Revisited. Ann. Oncol..

[64] Cloven N.G., Kyshtoobayeva A., Burger R.A., Yu I.R., Fruehauf J.P. (2004). In vitro chemoresistance and biomarker profiles are unique for histologic subtypes of epithelial ovarian cancer. Gynecol. Oncol..

[65] Itamochi H., Kigawa J., Terakawa N. (2008). Mechanisms of chemoresistance and poor prognosis in ovarian clear cell carcinoma. Cancer Sci..

[66] Xu W., Rush J., Rickett K., Coward J.I. (2016). Mucinous ovarian cancer: A therapeutic review. Crit. Rev. Oncol. Hematol..

[67] Tempfer C.B., Celik I., Solass W., Buerkle B., Pabst U.G., Zieren J., Strumberg D., Reymond M.-A. (2014). Activity of Pressurized Intraperitoneal Aerosol Chemotherapy (PIPAC) with Cisplatin and Doxorubicin in Women with Recurrent, Platinum-Resistant Ovarian Cancer: Preliminary Clinical Experience. Gynecol. Oncol..

[68] Tempfer C., Rezniczek G., Tsitas M., Ende P., Solass W., Demtroeder C., Reymond M. (2015). Pressurized intraperitoneal aerosol chemotherapy (PIPAC) with cisplatin and doxorubicin in 99 women with gynecologic malignancies and peritoneal carcinomatosis: A retrospective cohort study. Geburtshilfe Frauenheilkd..

[69] Tempfer C.B., Giger-Pabst U., Seebacher V., Petersen M., Dogan A., Rezniczek G.A. (2018). A Phase I, Single-Arm, Open-Label, Dose Escalation Study of Intraperitoneal Cisplatin and Doxorubicin in Patients with Recurrent Ovarian Cancer and Peritoneal Carcinomatosis. Gynecol. Oncol..

[70] Vizzielli G., Giudice M.T., Nardelli F., Costantini B., Salutari V., Inzani F.S., Zannoni G.F., Chiantera V., Di Giorgio A., Pacelli F. (2024). Pressurized IntraPeritoneal Aerosol Chemotherapy (PIPAC) Applied to Platinum-Resistant Recurrence of Ovarian Tumor: A Single-Institution Experience (ID: PARROT Trial). Ann. Surg. Oncol..

[71] Shi T., Jiang R., Yu J., Yang H., Tu D., Dai Z., Shen Y., Zhang Y., Cheng X., SGOG-OV/AICE Investigators (2018). Addition of Intraperitoneal Cisplatin and Etoposide to First-Line Chemotherapy for Advanced Ovarian Cancer: A Randomised, Phase 2 Trial. Br. J. Cancer.

[72] Monk B.J., Herzog T.J., Wang G., Triantos S., Maul S., Knoblauch R., McGowan T., Shalaby W.S.W., Coleman R.L. (2020). A Phase 3 Randomized, Open-Label, Multicenter Trial for Safety and Efficacy of Combined Trabectedin and Pegylated Liposomal Doxorubicin Therapy for Recurrent Ovarian Cancer. Gynecol. Oncol..

[73] Colombo N., Gadducci A., Sehouli J., Rulli E., Mäenpää J., Sessa C., Montes A., Ottevanger N.B., Berger R., Vergote I. (2023). INOVATYON/ENGOT-Ov5 Study: Randomized Phase III International Study Comparing Trabectedin/Pegylated Liposomal Doxorubicin (PLD) Followed by Platinum at Progression vs Carboplatin/PLD in Patients with Recurrent Ovarian Cancer Progressing within 6–12 Months after Last Platinum Line. Br. J. Cancer.

[74] Turinetto M., Ricotti A., Marchetti C., Pisano C., Zamagni C., Cassani C., Malaguti P., Baldoni A., Scollo P., Scandurra G. (2023). MITO39: Efficacy and Tolerability of Pegylated Liposomal Doxorubicin (PLD)–Trabectedin in the Treatment of Relapsed Ovarian Cancer after Maintenance Therapy with PARP Inhibitors—A Multicenter Italian Trial in Ovarian Cancer Observational Case-Control Study. Cancers.

[75] Nakka T., Goenka L., Dubashi B., Kayal S., Mathaiyan J., Barathi D., Krishnamoorthy N., Thumaty D.B., Dahagama S., Ganesan P. (2022). Phase II Study of Sodium Valproate in Combination with Oral Etoposide in Platinum-Resistant Ovarian Cancer. Med. Oncol..

[76] Zhou H., Liu Q., Zhang D., Li Q., Cao D., Cheng N., Wan X., Zhang Y., Feng F., Xiang Y. (2024). Efficacy and Safety of an Oral Combination Therapy of Niraparib and Etoposide in Platinum Resistant/Refractory Ovarian Cancer: A Single Arm, Prospective, Phase II Study. Int. J. Gynecol. Cancer.

[77] Matulonis U.A., Barry W., Penson R.T., Konstantinopoulos P.A., Luo W., Hoffman M.A., Horowitz N.S., Farooq S., Dizon D.S., Stover E. (2018). Phase II Study of Pembrolizumab (Pembro) Combined with Pegylated Liposomal Doxorubicin (PLD) for Recurrent Platinum-Resistant Ovarian, Fallopian Tube or Peritoneal Cancer. Gynecol. Oncol..

[78] Lee E.K., Xiong N., Cheng S.-C., Barry W.T., Penson R.T., Konstantinopoulos P.A., Hoffman M.A., Horowitz N., Dizon D.S., Stover E.H. (2020). Combined Pembrolizumab and Pegylated Liposomal Doxorubicin in Platinum Resistant Ovarian Cancer: A Phase 2 Clinical Trial. Gynecol. Oncol..

[79] Michels J., Ghiringhelli F., Frenel J.-S., Brard C., Genestie C., Balleyguier C., Ciccolini J., Paci A., You B., Floquet A. (2022). PemBOv Trial: Pembrolizumab plus Bevacizumab with or without Pegylated Liposomal Doxorubicin-Based Chemotherapy in Patients with Platinum-Resistant Ovarian Cancer. J. Clin. Oncol..

[80] Hamanishi J., Takeshima N., Katsumata N., Ushijima K., Kimura T., Takeuchi S., Matsumoto K., Ito K., Mandai M., Nakai H. (2021). Nivolumab Versus Gemcitabine or Pegylated Liposomal Doxorubicin for Patients With Platinum-Resistant Ovarian Cancer: Open-Label, Randomized Trial in Japan (NINJA). J. Clin. Oncol..

[81] Pujade-Lauraine E., Fujiwara K., Ledermann J.A., Oza A.M., Kristeleit R., Ray-Coquard I.-L., Richardson G.E., Sessa C., Yonemori K., Banerjee S. (2021). Avelumab Alone or in Combination with Chemotherapy versus Chemotherapy Alone in Platinum-Resistant or Platinum-Refractory Ovarian Cancer (JAVELIN Ovarian 200): An Open-Label, Three-Arm, Randomised, Phase 3 Study. Lancet Oncol..

[82] Fuh K.C., Bookman M.A., Liu J.F., Coleman R.L., Herzog T.J., Thaker P.H., Monk B.J., Anderson R., McIntyre G., Rangwala R. (2021). Phase 1b Study of AVB-500 in Combination with Paclitaxel or Pegylated Liposomal Doxorubicin Platinum-Resistant Recurrent Ovarian Cancer. Gynecol. Oncol..

[83] Wang T., Tang J., Yang H., Yin R., Zhang J., Zhou Q., Liu Z., Cao L., Li L., Huang Y. (2022). Effect of Apatinib Plus Pegylated Liposomal Doxorubicin vs Pegylated Liposomal Doxorubicin Alone on Platinum-Resistant Recurrent Ovarian Cancer: The APPROVE Randomized Clinical Trial. JAMA Oncol..

[84] Lan C.-Y., Wang Y., Xiong Y., Li J.-D., Shen J.-X., Li Y.-F., Zheng M., Zhang Y.-N., Feng Y.-L., Liu Q. (2018). Apatinib Combined with Oral Etoposide in Patients with Platinum-Resistant or Platinum-Refractory Ovarian Cancer (AEROC): A Phase 2, Single-Arm, Prospective Study. Lancet Oncol..

[85] Huang Q., Chu C., Tang J., Dai Z. (2020). Efficacy and Safety of Apatinib Combined with Etoposide in Patients with Recurrent Platinum-Resistant Epithelial Ovarian Cancer: A Retrospective Study. J. Cancer.

[86] Chen L., Luo J., Zhang J., Wang S., Sun Y., Liu Q., Cheng C. (2023). Dual Targeted Nanoparticles for the Codelivery of Doxorubicin and siRNA Cocktails to Overcome Ovarian Cancer Stem Cells. Int. J. Mol. Sci..

[87] Jin X., Zhou J., Zhang Z., Lv H. (2019). Doxorubicin Combined with Betulinic Acid or Lonidamine in RGD Ligand-Targeted pH-Sensitive Micellar System for Ovarian Cancer Treatment. Int. J. Pharm..

[88] Xia Y., Fang M., Dong J., Xu C., Liao Z., Ning P., Zeng Q. (2018). pH Sensitive Liposomes Delivering Tariquidar and Doxorubicin to Overcome Multidrug Resistance of Resistant Ovarian Cancer Cells. Colloids Surf. B Biointerfaces.

[89] Deshpande P., Jhaveri A., Pattni B., Biswas S., Torchilin V. (2018). Transferrin and Octaarginine Modified Dual-Functional Liposomes with Improved Cancer Cell Targeting and Enhanced Intracellular Delivery for the Treatment of Ovarian Cancer. Drug Deliv..

[90] Hashemi M., Ghadyani F., Hasani S., Olyaee Y., Raei B., Khodadadi M., Ziyarani M.F., Basti F.A., Tavakolpournegari A., Matinahmadi A. (2023). Nanoliposomes for Doxorubicin Delivery: Reversing Drug Resistance, Stimuli-Responsive Carriers and Clinical Translation. J. Drug Deliv. Sci. Technol..

[91] Han N.-N., Li X., Tao L., Zhou Q. (2018). Doxorubicin and Rhein Loaded Nanomicelles Attenuates Multidrug Resistance in Human Ovarian Cancer. Biochem. Biophys. Res. Commun..

[92] Tempfer C.B., Hilal Z., Dogan A., Petersen M., Rezniczek G.A. (2018). Concentrations of Cisplatin and Doxorubicin in Ascites and Peritoneal Tumor Nodules before and after Pressurized Intraperitoneal Aerosol Chemotherapy (PIPAC) in Patients with Peritoneal Metastasis. Eur. J. Surg. Oncol..

[93] Taliento C., Restaino S., Scutiero G., Arcieri M., Bernardi G., Martinello R., Driul L., Perrone A.M., Fagotti A., Scambia G. (2023). Pressurized Intraperitoneal Aerosol Chemotherapy (PIPAC) with Cisplatin and Doxorubicin in Patients with Ovarian Cancer: A Systematic Review. Eur. J. Surg. Oncol..

[94] Boccia S.M., Sassu C.M., Ergasti R., Vertechy L., Apostol A.I., Palluzzi E., Fagotti A., Scambia G., Marchetti C. (2024). Focus on Trabectedin in Ovarian Cancer: What Do We Still Need to Know?. Drug Des. Dev. Ther..

[95] Gottlicher M. (2001). Valproic Acid Defines a Novel Class of HDAC Inhibitors Inducing Differentiation of Transformed Cells. EMBO J..

[96] Kwiecińska P., Taubøll E., Grzyb E., Fiedor E., Ptak A., Gregoraszczuk E.L. (2016). Valproic Acid as a Promising Co-Treatment With Paclitaxel and Doxorubicin in Different Ovarian Carcinoma Cell Lines. Int. J. Gynecol. Cancer.

[97] Eetezadi S., Evans J.C., Shen Y.-T., De Souza R., Piquette-Miller M., Allen C. (2018). Ratio-Dependent Synergism of a Doxorubicin and Olaparib Combination in 2D and Spheroid Models of Ovarian Cancer. Mol. Pharm..

[98] Perez-Fidalgo J.A., Iglesias M., Bohn U., Calvo E., Garcia Y., Guerra E., Manso L., Santaballa A., Gonzalez-Martin A. (2019). GEICO1601-ROLANDO: A Multicentric Single Arm Phase II Clinical Trial to Evaluate the Combination of Olaparib and Pegylated Liposomal Doxorubicin for Platinum-Resistant Ovarian Cancer. Future Sci. OA.

[99] Madariaga A., Coleman R.L., González Martín A. (2023). Novel Therapies Leading to a New Landscape in Gynecologic Tumors. Int. J. Gynecol. Cancer.

[100] Peng H., He X., Wang Q. (2022). Immune Checkpoint Blockades in Gynecological Cancers: A Review of Clinical Trials. Acta Obstet. Gynecol. Scand..

[101] Mauricio D., Zeybek B., Tymon-Rosario J., Harold J., Santin A.D. (2021). Immunotherapy in Cervical Cancer. Curr. Oncol. Rep..

[102] Turinetto M., Valsecchi A.A., Tuninetti V., Scotto G., Borella F., Valabrega G. (2022). Immunotherapy for Cervical Cancer: Are We Ready for Prime Time?. Int. J. Mol. Sci..

[103] Grau J.-F., Farinas-Madrid L., Garcia-Duran C., Garcia-Illescas D., Oaknin A. (2023). Advances in Immunotherapy in Cervical Cancer. Int. J. Gynecol. Cancer.

[104] Marín-Jiménez J.A., García-Mulero S., Matías-Guiu X., Piulats J.M. (2022). Facts and Hopes in Immunotherapy of Endometrial Cancer. Clin. Cancer Res..

[105] Mahdi H., Chelariu-Raicu A., Slomovitz B.M. (2023). Immunotherapy in Endometrial Cancer. Int. J. Gynecol. Cancer.

[106] Bogani G., Monk B.J., Powell M.A., Westin S.N., Slomovitz B., Moore K.N., Eskander R.N., Raspagliesi F., Barretina-Ginesta M.-P., Colombo N. (2024). Adding Immunotherapy to First-Line Treatment of Advanced and Metastatic Endometrial Cancer. Ann. Oncol..

[107] Martinez-Cannon B.A., Colombo I. (2024). The Evolving Role of Immune Checkpoint Inhibitors in Cervical and Endometrial Cancer. Cancer Drug Resist..

[108] Stefanoudakis D., Karopoulou E., Matsas A., Katsampoula G.A., Tsarna E., Stamoula E., Christopoulos P. (2024). Immunotherapy in Cervical and Endometrial Cancer: Current Landscape and Future Directions. Life.

[109] Borella F., Ghisoni E., Giannone G., Cosma S., Benedetto C., Valabrega G., Katsaros D. (2020). Immune Checkpoint Inhibitors in Epithelial Ovarian Cancer: An Overview on Efficacy and Future Perspectives. Diagnostics.

[110] Leary A., Tan D., Ledermann J. (2021). Immune Checkpoint Inhibitors in Ovarian Cancer: Where Do We Stand?. Ther. Adv. Med. Oncol..

[111] Indini A., Nigro O., Lengyel C.G., Ghidini M., Petrillo A., Lopez S., Raspagliesi F., Trapani D., Khakoo S., Bogani G. (2021). Immune-Checkpoint Inhibitors in Platinum-Resistant Ovarian Cancer. Cancers.

[112] Pawłowska A., Rekowska A., Kuryło W., Pańczyszyn A., Kotarski J., Wertel I. (2023). Current Understanding on Why Ovarian Cancer Is Resistant to Immune Checkpoint Inhibitors. Int. J. Mol. Sci..

[113] Ghisoni E., Morotti M., Sarivalasis A., Grimm A.J., Kandalaft L., Laniti D.D., Coukos G. (2024). Immunotherapy for Ovarian Cancer: Towards a Tailored Immunophenotype-Based Approach. Nat. Rev. Clin. Oncol..

[114] Rankin E.B., Fuh K.C., Castellini L., Viswanathan K., Finger E.C., Diep A.N., LaGory E.L., Kariolis M.S., Chan A., Lindgren D. (2014). Direct Regulation of GAS6/AXL Signaling by HIF Promotes Renal Metastasis through SRC and MET. Proc. Natl. Acad. Sci. USA.

